# Order Concerning the Importation of Cattle from the Republic of Mexico

**Published:** 1895-11

**Authors:** J. Sterling Morton

**Affiliations:** Secretary; U. S. Department of Agriculture, Office of the Secretary, Washington, D. C.


### Abstracts and Selections.

### Order Concerning the Importation of Cattle from the Republic of Mexico.

U. S. Department of Agriculture,
Office of the Secretary,
Washington, D. C., September 27, 1895.

Pursuant to the authority vested in the Secretary of Agriculture by virtue of an act of Congress, entitled "An act for the establishment of a Bureau of Animal Industry, to prevent the exportation of diseased cattle, and to provide means for the suppression and extirpation of pleuro-pneumonia and other contagious diseases among domestic animals," approved May 29, 1884, and of an act entitled "An act providing for the inspection of meats for exportation, prohibiting the importation of adulterated articles of food or drink, authorizing the President to make proclamation in certain cases, and for other purposes," approved August 30, 1890, and also of an act entitled "An act making appropriations for the Department of Agriculture for the fiscal year ending June 30, 1896," approved March 2, 1895, it is ordered that from and after October 22, 1895, cattle may be admitted into the United States from the Republic of Mexico, for grazing and for immediate slaughter, through the ports of San Diego, Nogales, El Paso, Eagle Pass, Brownsville, and the sub-port of Laredo. The admission of said cattle is permitted subject to inspection by an inspector of the Bureau of Animal Industry; and no cattle will be admitted which are affected with or which have been exposed to the contagion of any disease liable to be disseminated among the domestic animals of the United States. The importer must produce evidence satisfactory to the inspector, that his cattle have not been exposed to contagion during a period of ninety days previous to the importation.

Cattle imported into the district of the United States known as the Texas or splenetic fever district, will be subject to all regulations applying to the native cattle of that district. Cattle imported into other sections of the United States previous to December 1, 1895, must have been held for three months in the elevated districts, free from Texas or splenetic fever infection, of the States of Sonora and Chihuahua, and will be subject to the laws and rules and regulations of the States and Territories into which they are taken, and also subject to such regulations of this Department as may, from time to time, be made. The formal entry of the cattle and the application for inspection must be made at the places mentioned in this order, but the cattle will be allowed to cross the boundary at such points as may be agreed upon by the collector of customs of the district and the inspector of this department as proper and convenient for inspection.

The operation of all orders and of all regulations, or parts of regulations, inconsistent with this order, is hereby suspended until further notice.

J. STERLING MORTON, Secretary.

